# Contact varroacidal efficacy of lithium citrate and its influence on viral loads, immune parameters and oxidative stress of honey bees in a field experiment

**DOI:** 10.3389/fphys.2022.1000944

**Published:** 2022-09-12

**Authors:** Nemanja M. Jovanovic, Uros Glavinic, Marko Ristanic, Branislav Vejnovic, Jevrosima Stevanovic, Milivoje Cosic, Zoran Stanimirovic

**Affiliations:** ^1^ Department of Parasitology, Faculty of Veterinary Medicine, University of Belgrade, Belgrade, Serbia; ^2^ Department of Biology, Faculty of Veterinary Medicine, University of Belgrade, Belgrade, Serbia; ^3^ Department of Economics and Statistics, Faculty of Veterinary Medicine, University of Belgrade, Belgrade, Serbia; ^4^ Institute of Forestry, Belgrade, Serbia; ^5^ Department of Animal Breeding, Faculty of Agriculture, Bijeljina University, Bijeljina, Bosnia and Herzegovina

**Keywords:** lithium citrate, *Varroa destructor*, *Apis mellifera*, viral load, immune-related genes, oxidative stress

## Abstract

With an almost global distribution, *Varroa destuctor* is the leading cause of weakening and loss of honey bee colonies. New substances are constantly being tested in order to find those that will exhibit high anti-*Varroa* efficacy at low doses/concentrations, without unwanted effects on bees. Lithium (Li) salts stood out as candidates based on previous research. The aims of this study were to evaluate Li citrate hydrate (Li-cit) for its contact efficacy against *Varroa*, but also the effect of Li-cit on honey bees by estimating loads of honey bee viruses, expression levels of immune-related genes and genes for antioxidative enzymes and oxidative stress parameters on two sampling occasions, before the treatment and after the treatment. Our experiment was performed on four groups, each consisting of seven colonies. Two groups were treated with the test compound, one receiving 5 mM and the other 10 mM of Li-cit; the third received oxalic acid treatment (OA group) and served as positive control, and the fourth was negative control (C group), treated with 50% w/v pure sucrose-water syrup. Single trickling treatment was applied in all groups. Both tested concentrations of Li-cit, 5 and 10 mM, expressed high varroacidal efficacy, 96.85% and 96.80%, respectively. Load of Chronic Bee Paralysis Virus significantly decreased (*p* < 0.01) after the treatment in group treated with 5 mM of Li-cit. In OA group, loads of Acute Bee Paralysis Virus and Deformed Wing Virus significantly (*p* < 0.05) increased, and in C group, loads of all viruses significantly (*p* < 0.01 or *p* < 0.001) increased. Transcript levels of genes for abaecin, apidaecin, defensin and vitellogenin were significantly higher (*p* < 0.05—*p* < 0.001), while all oxidative stress parameters were significantly lower (*p* < 0.05—*p* < 0.001) after the treatment in both groups treated with Li-cit. All presented results along with easy application indicate benefits of topical Li-cit treatment and complete the mosaic of evidence on the advantages of this salt in the control of *Varroa*.

## Introduction

The honey bee mite *V. destructor* (Acari: Varroidae), is considered the most damaging parasite of the honey bee *A. mellifera* L. (Carreck et al., 2010; Guzmán-Novoa et al., 2010; Le Conte et al., 2010; Jack and Ellis 2021). Being distributed nearly all around the world, *Varroa* is the leading cause of honey bee colony weakening and loss (Genersch et al., 2010; Rosenkranz et al., 2010; Stanimirović et al., 2019, 2022a; Noël et al., 2020; Traynor et al., 2020; Boncristiani et al., 2021). As an obligate ectoparasite of *A. mellifera* bees, *Varroa* causes serious health problems of the host due to feeding on the fat body and haemolymph of adult and developing honey bees (Rosenkranz et al., 2010; Ramsey et al., 2019) and the role of mechanical or biological vector of pathogene microorganisms, especially viruses, with a potencial to exacerbate preexisting infections (Genersch and Aubert, 2010; Glavinić et al., 2014; McMenamin and Genersch 2015; Noël et al., 2020; Traynor et al., 2020). Deformed Wing virus (DWV), Acute Bee Paralysis Virus (ABPV), Chronic Bee Paralysis Virus (CBPV) and Sacbrood Virus (SBV) are considered risk factors for honey bee colony health (Martin et al., 2012) and are globally distributed, often without clinical signs (Cirkovic et al., 2018). Combination of *Varroa* and DWV is particularly threatening to honey bee health due to its negative effect on immunity and longevity of bees (Martin et al., 2012; Nazzi et al., 2012; Schöning et al., 2012; Di Prisco et al., 2016; Zhao et al., 2019). Negative influence on honey bees is also reported for*d Varroa*-ABPV combination (Genersch and Aubert 2010; Genersch et al., 2010; Gregorc et al., 2012; Francis et al., 2013).

One of the basic prerequisites for maintaining healthy and strong bee colonies is monitoring and controlling the level of *Varroa* infestation. Several different methods of controlling *Varroa* populations have been developed. Beside the application of biotechnical control methods, chemical control is the most important and is reflected in its use of “hard” (synthetic active ingredients) and “soft” (natural-based active ingredients) acaricides (Stanimirović et al., 2019). The use of synthetic acaricides ensures high antivarosis efficacy. However, *Varroa* resistance to these acaricides (Loucif-Ayad et al., 2010; Maggi et al., 2010; Gonzalez-Cabrera et al., 2013, 2016; Kamler et al., 2016; Beaurepaire et al., 2017; Stara et al., 2019) and the occurrence of acaricide residues in bee products (Mullin et al., 2010; Wu et al., 2011; Sanchez-Bayo and Goka 2014; Ostiguy et al., 2019) are limiting factors in their application. Also, numerous studies have described the side effect of “hard” acaricide residues on the health of bees (Johnson et al., 2009; Boncristiani et al., 2012; Medici et al., 2012; Wu et al., 2011; Berry et al., 2013; Johnson et al., 2013a; Johnson et al., 2013b; Williamson and Wright 2013). Therefore, the application of “soft” preparations in the control of *Varroa* is an important factor in beekeeping due to the lower risk of resistance and residues in the hive (Rosenkranz et al., 2010). Many natural preparations have shown an anti-*Varroa* efficacy, with organic acids (primarily formic and oxalic acid), essential oils (most of which are thymol-based) and beta acids from hops (*Humulus lupulus*) being the most tested and used to make commercial formulations (Rademacher et al., 2015; Maggi et al., 2016; Brasesco et al., 2017; Stanimirovic et al., 2017; Conti et al., 2020; Rashid et al., 2020). However, the efficacy of natural-based acaricides is variable (Rosenkranz et al., 2010; Pietropaoli & Formato, 2018, 2019; Jack et al., 2021a,b) and both organic acids (particulary oxalic acid) and essential oils (particularly thymol and menthol) may be toxic and harm all three castes of honey bees and all developmental stages (Marchetti et al., 1984; Ellis and Baxendale, 1997; Rice et al., 2002; Floris et al., 2004; Ebert et al., 2007; Damiani et al., 2009; Gregorc et al., 2018). Finally, the use of one strategy/method does not provide a permanent solution, so it is necessary to combine several strategies in order to achieve adequate results in *Varroa* combat. For example, diet supplements may indirectly contribute in *Varroa* control by enhancing hygienic behaviour (Stanimirovic et al., 2022a) that reflected in successful reduction of viral infections. New substances have been continuously sought for decades in order to find those that will exhibit high varroacidal efficacy at low doses/concentrations, without side effects on bees (Stanimirović et al., 2019; Jack and Ellis 2021). Several studies have shown that lithium (Li) salts are good candidates for this purpose (Ziegelmann et al., 2018; Kolics et al., 2020, 2021a,b, 2022; Stanimirovic et al., 2022b; Sevin et al., 2022). The varroacidal effect of Li salts was first described by Ziegelmann et al. (2018) in which bees were fed different Li salts under both laboratory and field conditions (but on artificial swarms). Different concentrations of Li chloride and Li citrate (in the range of 2–25 mM) showed a remarkable anti-*Varroa* effect, with both salts being well tolerated by the worker bees (Ziegelmann et al., 2018). For Li chloride, in addition to the systemic effect, a contact efficacy against *Varroa* was also proven on bee colonies, whereby the effectiveness of the mentioned salt applied to paperboard strips was 100% (Kolics et al., 2020). In another study by these authors (Kolics et al., 2021a) in the field experiment, the effectiveness of Li chloride and oxalic acid was tested in parallel; the efficacy of Li chloride (applied by trickling) was 95.6%. In our previous study (Stanimirovic et al., 2022b), systemic effect of two Li salts (Li chloride and Li citrate) were evaluated in laboratory conditions by oral administration of six concentrations (in a range from 1 to 25 mM). Li citrate hydrate (Li-cit) in concentrations of 4 and 7.5 mM showed the best anti-*Varroa* activity (100%) and best honey bee tollerance (100% of bees survived). Consequently, in field trials (on full-sized hives), Stanimirovic et al. (2022b) tested only Li-cit. Concentrations in a range from 5 to 25 mM achieved varroacidal efficacy 93.2%–95.5%. Beside systemic acaricide effect of Li-cit, Li residues were measured in hive products. None of the applied Li-cit concentrations resulted in significant increase of Li residues in wax; the residues were even significantly lower in wax combs and wax cappings compared to commercial wax foundations (Stanimirovic et al., 2022b). Similarly, in a work where colonies received 25 mM of Li chloride, wax remained Li-free (Kolics et al., 2021b). Honey originated from brood chamber was not affected with lower concentrations (15 and 20 mM) of Li-cit (Stanimirovic et al., 2022b), so concentrations in a range from 4 to 10 mM were declared promising (Stanimirovic et al., 2022b) and taken into account for contact efficacy testing in this study. When it comes to the method of application, we considered results of Kolics et al. (2020, 2021a, 2022) who concluded that trickling method is the most effective way of administration of Li chloride.

The challenge for the invention and development of new methods in the control of the *Varroa* continues. Based on already reported anti-*Varroa* effects of tested Li salts, the aim of this study was to evaluate contact varroacidal efficacy of Li-cit treatment (carried out by trickling) in a field experiment, along with the assessment of treatment effects on honey bees by analyzing load of honey bee viruses, expression of genes important for immunity and those for antioxidant enzymes, and oxidative stress parameters.

## Materials and methods

### Chemicals

Li-cit was the test substance (97%, CAS No. 313222-91-2, Sigma Aldrich). It was dissolved in 1,000 ml of 50% w/v sucrose-water syrup to obtain two final concentrations, 5 and 10 mM. For positive control group, 3% solution of oxalic acid dihydrate (OA) was used (CAS No. 144-62-7) after dissolving 30 g of OA in 1,000 ml of 50% w/v sucrose-water syrup. Follow-up treatments were done using CheckMite+^®^ (Bayer, Healthcare AG), the most efficient registered varroacide in Serbia, probably because it is the least used of all available varroacides (as documented by a recent work of Brodschneider et al., 2022) so *Varroa* populations have not developed resistance to it. This “hard” acaricide showed 96%–97% varroacidal efficacy in Serbia (Zikic et al., 2020) and 91.70% efficacy in neighboring country Croatia (Tlak-Gajger and Susec, 2019), it is chemically unrelated to the test substance, thus fulfilling all requirements of European Medicines Agency (EMA, 2021) for follow-up treatment.

### Experimental design

**FIGURE 1:**
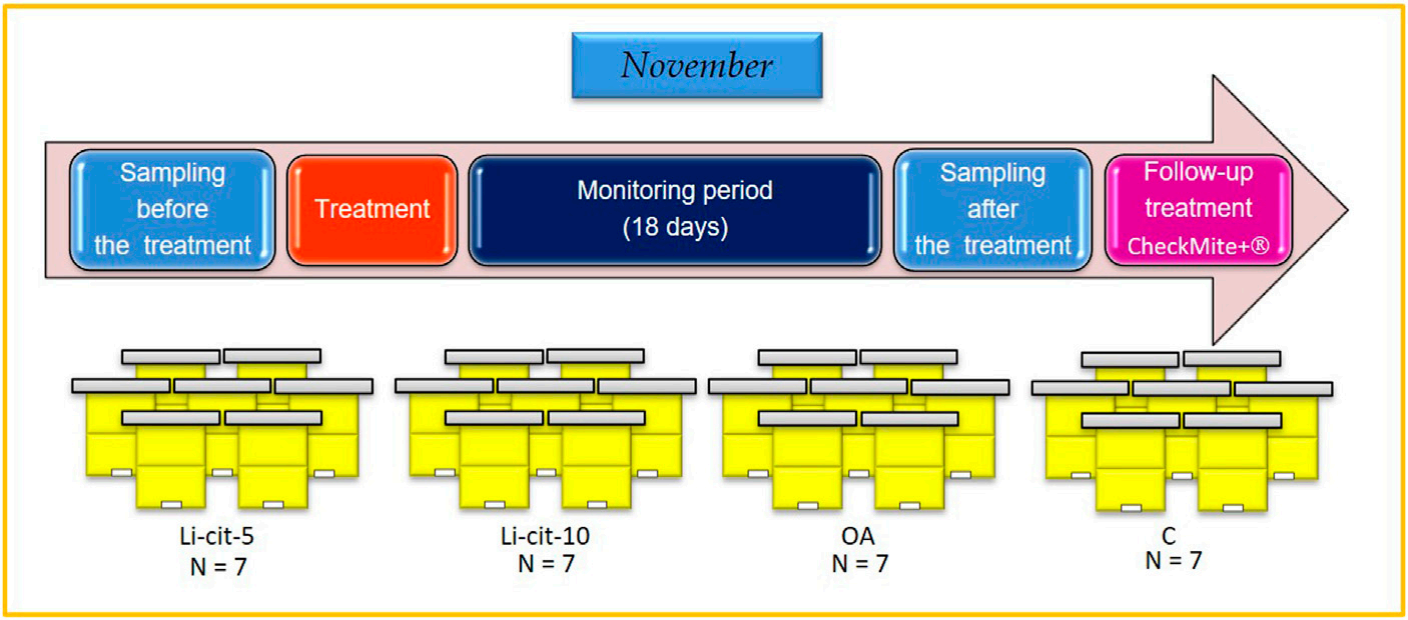
Experimental design. Li-cit-5, group treated with 5 mM of lithium citrate; Li-cit-10, group treated with 10 mM of lithium citrate; OA, group treated with oxalic acid (positive control); C, negative control.

Honey bee colonies used in the experiment were located in Podnemic village (Ljubovija municipality, 44°10′24.6″N; 19°24′38.0″E) in western Serbia. During the period of preparing for wintering, in the summer of 2020, the colonies were uniformed in respect of their strength parameters (open brood area, sealed brood area, reserves of stored honey and pollen/bee bread, adult bee population) (Delaplane et al., 2013; Jovanovic et al., 2021). Colonies were regularly checked for both bee and brood pathology by a veterinary specialist (authors of this work), and no disease, except varroosis, was affirmed. In November 2020, highly infested colonies were selected (≥20 naturally fallen *Varroa* mites/day) and four experimental groups were established with seven colonies per group. Each colony in the experiment represented one experimental unit. Two groups were intended for the treatment with Li-cit, first one with 5 mM (Li-cit-5 group) and another with 10 mM (Li-cit-10 group); third group was planned to receive OA treatment (OA group) and to serve as positive control, while the fourth was negative control (C group), treated with 50% w/v pure sucrose-water syrup (Figure 1).

### Estimation of varroacidal efficacy

For estimation of the mite fall number, each experimental hive was equipped with screened (“anti-*Varroa*”) bottom board with insert covered with neutral oil to be sticky (Stanimirovic et al., 2017). Treatments were applied by trickling a syrup onto the adult bees between frames (5 ml of solution per bee space) with a 50 ml syringe. Colonies were monitored over a period of 18 days, and fallen mites were collected and counted every day. After 18 days, follow-up treatment was performed using CheckMite+^®^, two strips per colony for 6 weeks (as recommended by the producer). Varroacide efficacy of test treatments and OA was calculated according to the formula: No. of mites killed by test compound × 100/(No. of mites killed by test compound + No. of mites killed after follow-up treatment) we previously used (Stanimirovic et al., 2022b), but also adopted by European Medicines Agency (EMA, 2021). In negative control, natural mite fall was monitored.

### Sampling of bees

Seventy adult worker bees from each colony were sampled on two sampling occasions: first, before the treatment (BT) and second, after the treatment (AT)—on 18th day of the experiment. From each sample, 40 bees were used for viral load estimations and gene expression analyses and the rest of 30 bees served for analyses of oxidative stress parameters. Bees were taken from peripheral frames, frozen in dry ice and transported to the laboratory where they were stored at −80°C until analysis.

### Virus load estimation and gene expression quantification

Each sample, consisting of 40 bees, was macerated and homogenized in 6 ml of PBS. Total RNA was extracted using Quick-RNA MiniPrep Kit (Zymo Research), following the guidelines of manufacturer. RNA extraction process included “in-column DNase treatment” (treatment with DNase I reaction mixture) aimed to remove the remaining DNA. Samples were equalized to contain 1,000 ng of RNK in 1µL. The extracted RNA was immediately converted into cDNA using RevertAid™ First Strand cDNA Synthesis Kit (Fermentas), in accordance with the manufacturer’s protocol and the obtained cDNA was stored at −20°C until further analysis.

For quantitative real time PCR (qPCR) with SYBR green technique, KAPA SYBR1 FAST Master Mix (2X) Universal set (KAPA Biosystems) was used (according to the protocol of manufacturer) and 20-μl reactions were processed in “Rotor-Gene Q 5plex” (Qiagen). Primer used are presented in Supplementary Table S1. Load of ABPV, CBPV, DWV, and SBV were estimated. Viral quantities were determined using the comparative ΔCt method. Briefly, the average quantity of each virus was optimized according to the Ct value of the ‘housekeeping’ gene for β-actin (ΔCt = Ct_virus_−Ct_β-actin_) as described in Jovanovic et al. (2021).

Transcript levels of genes for abaecin, apidaecin, defensin, hymenoptaecin, vitellogenin, Cu/Zn superoxide dismutase (Cu/ZnSOD), Mn superoxide dismutase (MnSOD), catalase (CAT), glutathione S-transferase (GST) were determined. Quantification of gene expression levels was done using 2^−ΔCt^ method. For normalization of each gene expression β-actin was used as an internal control gene (described in Glavinic et al., 2021b).

### Analyses of oxidative stress parameters

In each sample, consisting of 30 bees, following oxidative stress parameters were assessed: activities of three antioxidative enzymes: superoxide dismutase (SOD), catalase (CAT), and glutathione S-transferase (GST), and concentration of malondialdehyde (MDA). Analyses were done by UV/VIS Spectrophotometer BK-36 S390 (Biobase) with methodology described in Taric et al. (2020) and Zikic et al. (2020).

### Statistical analysis

Data for varroacidal efficacy, load of viruses (ABPV, CBPV, DWV, and SBV), expression level of genes (for abaecin, apidaecin, defensin, hymenoptaecin, vitellogenin, GST, MnSOD, CuZnSOD and CAT) and values of oxidative stress parameters (SOD, CAT, and GST activities and MDA concentration) were tested for normality by using Shapiro–Wilk’s test. Given that data for viral loads and gene expression levels were not normally distributed (Shapiro–Wilk’s test, *p* < 0.05) adequate transformations were made: viral loads were expressed as log10 and for the gene expression levels, log10(y + 10) transformation was applied. For data on varroacidal efficacy, groups were compared in one-way ANOVA followed by Tukey’s test. For viral loads, gene expression levels and values of oxidative stress parameters, groups were compared in two-way ANOVA with repeated measures in one factor, followed by Tukey’s test between groups for each sampling occasion (BT and AT) and Sidak’s test between sampling occasions (between BT and AT) in each group. Significant difference was estimated at *p* < 0.05, *p* < 0.01 and *p* < 0.001 significance levels. Statistical analysis of the results obtained in the experiment was carried out using statistical software GraphPad Prism version 7 (GraphPad, San Diego, CA, United States).

## Results

### Varroacidal efficacy

**FIGURE 2:**
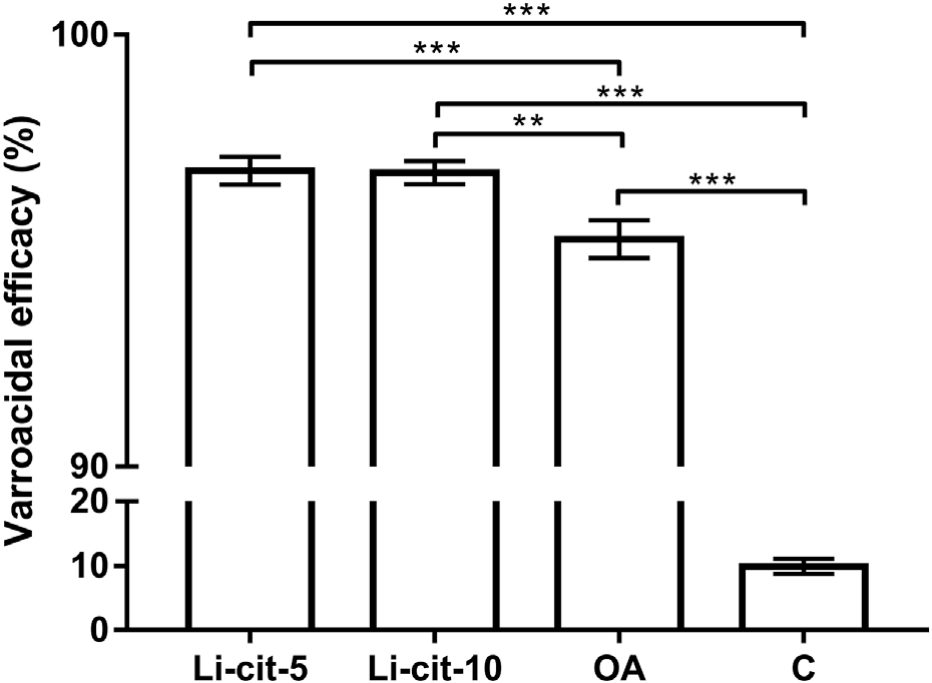
Varroacidal efficacy (%) in two Li-cit groups (Li-cit-5 and Li-cit-10) compared to groups OA and C. Bars indicate mean ± standard deviation. **p < 0.01; ***p < 0.001; Li-cit-5, group treated with 5 mM of lithium citrate; Li-cit-10, group treated with 10 mM of lithium citrate; OA, group treated with oxalic acid (positive control); C, negative control.

Both tested concentrations of Li-cit, 5 and 10 mM, showed high efficacy against *Varroa* (96.85% and 96.80%, respectively) and those efficacies were significantly higher (*p* < 0.001 and *p* < 0.01, respectively) compared to OA group, and to C group (*p* < 0.001 for both concentrations). The efficacy of oxalic acid was also high (95.30%) and significantly higher (*p* < 0.001) than natural mite fall in C group (negative control) (Figure 2).

### Viral loads

**FIGURE 3:**
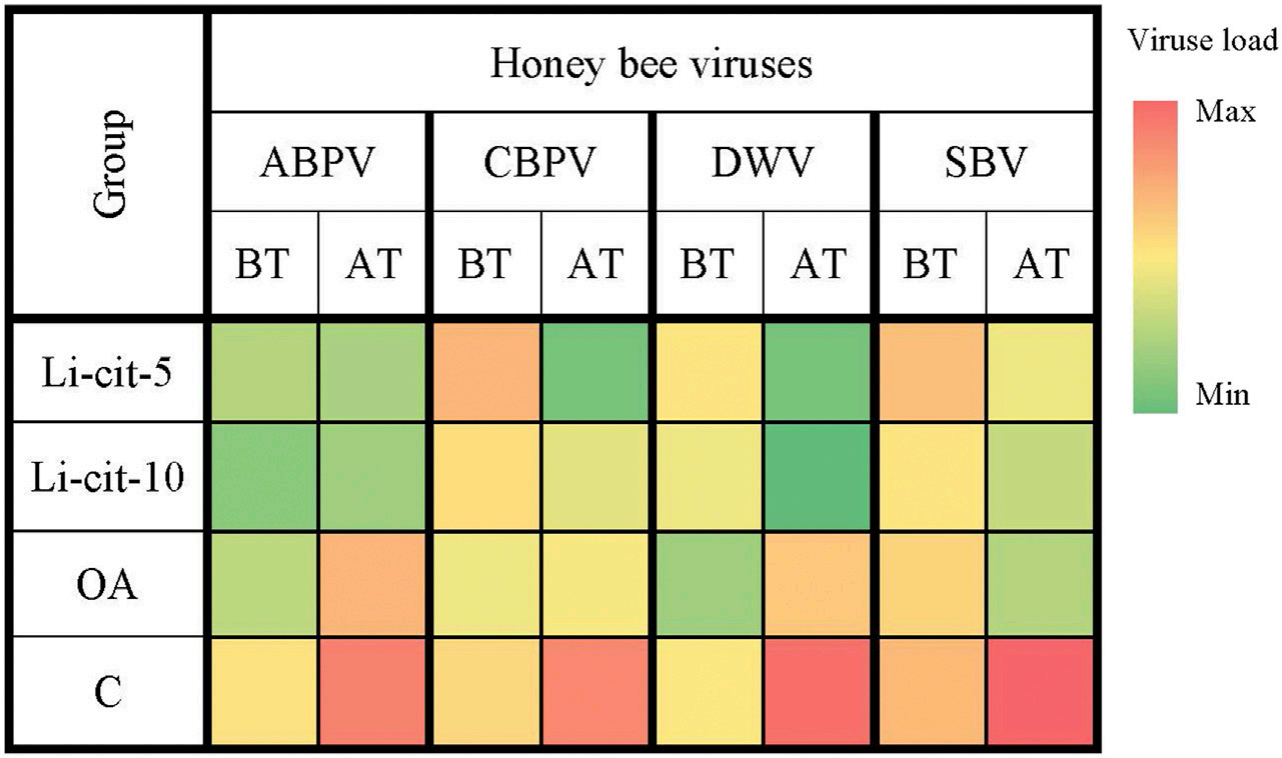
Heatmap: Loads of honey bee viruses (means of Log10 Ct values) in different sampling occasions (before and after the treatment) within each group. ABPV, Acute Bee Paralysis Virus; CBPV, Chronic Bee Paralysis Virus; DWV, Deformed Wing Virus; SBV, Sacbrood Virus; BT, before the treatment; AT, after the treatment; Li-cit-5, group treated with 5 mM of lithium citrate; Li-cit-10, group treated with 10 mM of lithium citrate; OA, group treated with oxalic acid (positive control); C, negative control.

The loads of ABPV and DWV in groups Li-cit-5 and Li-cit-10 were not significantly different between two sampling occasions (before and after the treatment), but in groups OA and C the infection of both viruses significantly (*p* < 0.01 and *p* < 0.001, respectively) increased over time (Figure 3, Supplementary Figures S1, S3). Analyzing the obtained data between groups after the treatment, a significantly lower (*p* < 0.01 or *p* < 0.001) load of ABPV and DWV was recorded in groups treated with Li-cit compared to the OA and C groups (Figure 3, Supplementary Figures S1, S3). A significantly lower (*p* < 0.001) load of DWV was recorded in the OA group compared to the C group (Figure 3, Supplementary Figures S1, S3).

CBPV load in group Li-cit-5 significantly decreased (*p* < 0.01) over time, while in group C, it (*p* < 0.01) significantly increased (Figure 3, Supplementary Figure S2). When CBPV loads were compared between groups, significant (*p* < 0.01) difference was found between Li-cit-5 and OA groups before the treatment, while after the treatment, the highest CBPV load was in C group and was significantly higher (*p* < 0.001) compared to Li-cit-5, Li-cit-10 and OA groups (Figure 3, Supplementary Figure S2).

Load of SBV in C group significantly increased (*p* < 0.01) over time and after the treatment was also significantly higher (*p* < 0.001) compared to all other groups (Figure 3, Supplementary Figure S4).

### Expression of immune-related genes and antioxidant enzyme genes

**FIGURE 4:**
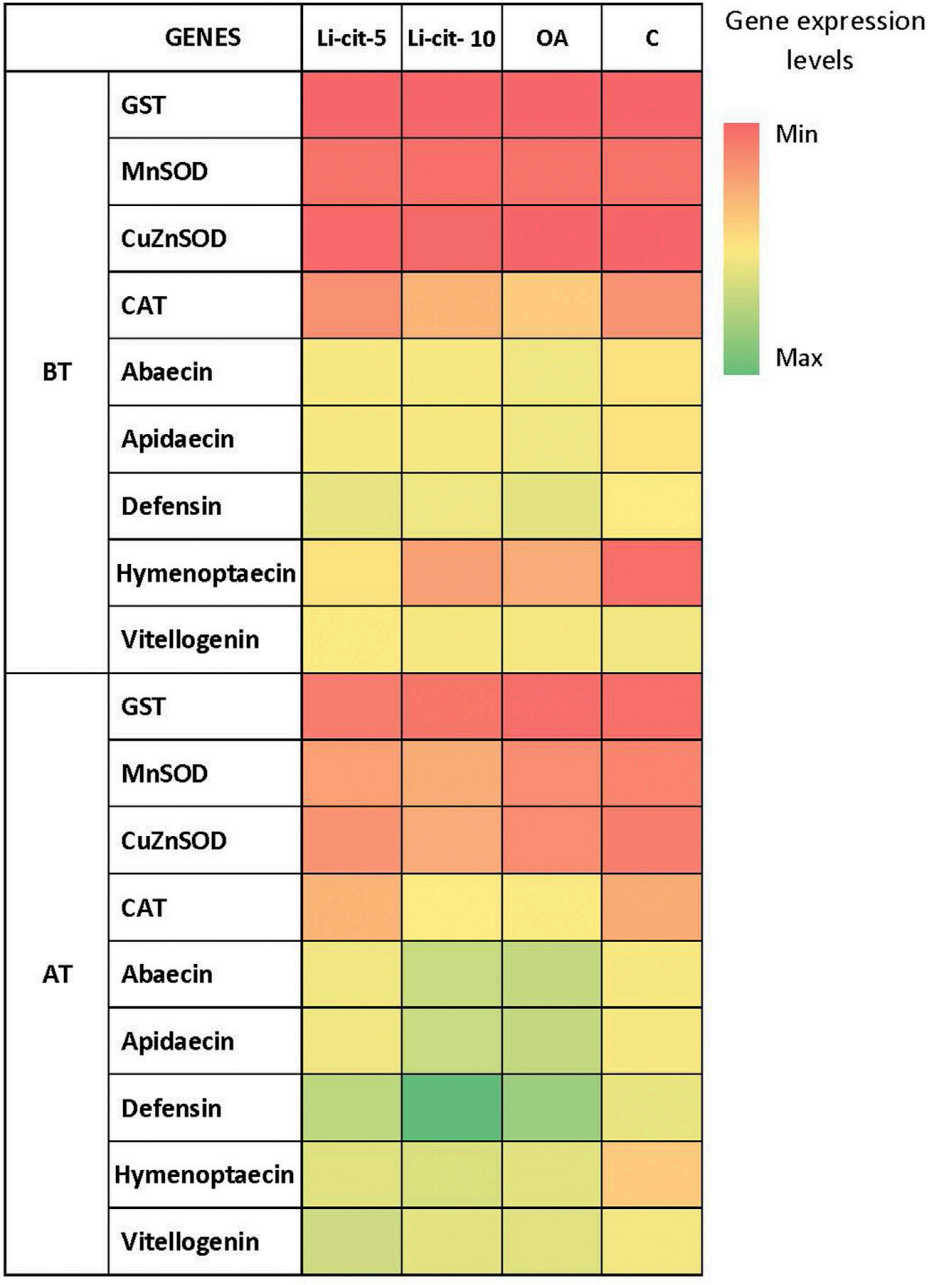
Heatmap: Expression levels [means of Log10 (y + 10)] of immune-related genes (abaecin, apidaecin, defensin, hymenoptaecin and vitellogenin) and genes for antioxidative enzymes (GST, CuZnSOD, MnSOD, CAT) in different sampling occasions (before and after the treatment) within each group. GST, glutathione S-transferase, CuZnSOD, CuZn superoxide dismutase, MnSOD, Mn superoxide dismutase, CAT, catalase); BT, before the treatment; AT, after the treatment; Li-cit-5, group treated with 5 mM of lithium citrate; Li-cit-10, group treated with 10 mM of lithium citrate; OA, group treated with oxalic acid (positive control); C, negative control.

After the treatment, expression levels were significantly higher compared to pre-treatment values in case of: Vitellogenin and defensin genes (*p* < 0.01) in Li-cit-5 group; abaecin, apidaecin and defensin genes (*p* < 0.01 or *p* < 0.001) in Li-cit-10 group; abaecin and apidaecin genes (*p* < 0.05) in OA group. Only in C group, none of the genes changed the expression between two sampling occasions (Figure 4).

No differences (*p* > 0.05) were found in transcript levels of GST, CuZnSOD, MnSOD and CAT genes (Figure 4) in Li-cit-5, Li-cit-10 and OA groups between two sampling occasions. Only in C group, expression levels of CuZnSOD, MnSOD and GST genes were significantly higher (*p* < 0.05 or *p* < 0.01) after the treatment compared to the values recorded before the treatment.

### Oxidative stress parameters

**FIGURE 5:**
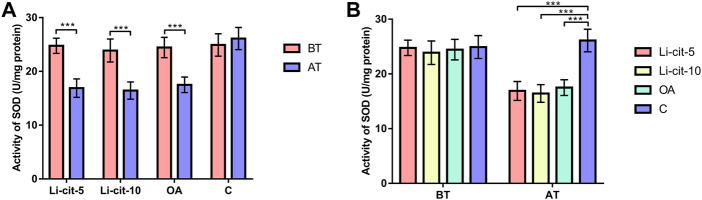
Superoxide dismutase activity: comparisons between sampling occasions (before and after the treatment) within each group **(A)** and comparisons between groups at each sampling occasion **(B)**. Bars indicate mean ± standard deviation. **p < 0.01; ***p < 0.001; BT, before the treatment; AT, after the treatment; Li-cit-5, group treated with 5 mM of lithium citrate; Li-cit-10, group treated with 10 mM of lithium citrate; OA, group treated with oxalic acid (positive control); C, negative control.

**FIGURE 6:**
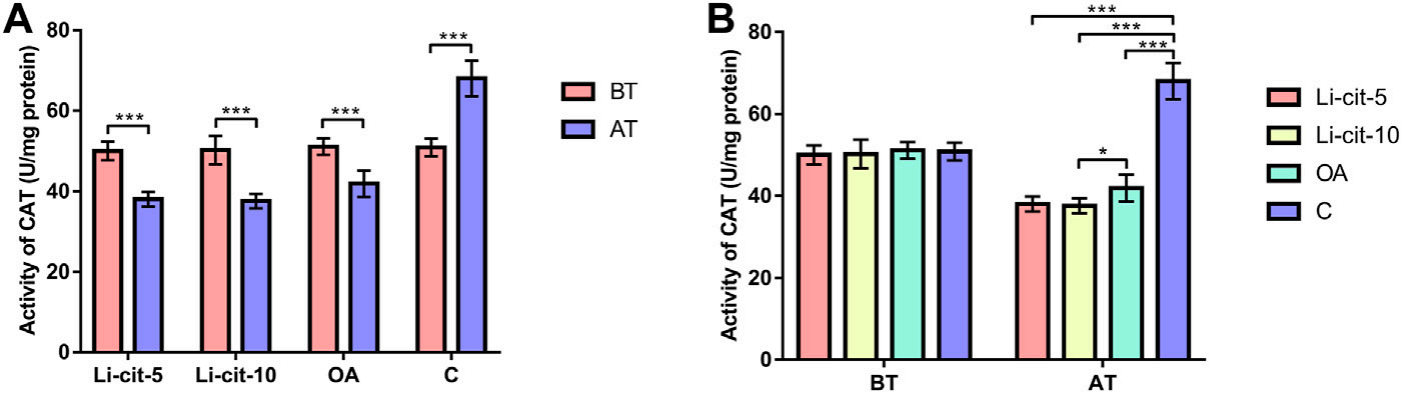
Catalase activity: comparisons between sampling occasions (before and after the treatment) within each group **(A)** and comparisons between groups at each sampling occasion **(B)**. Bars indicate mean ± standard deviation. *p < 0.05; ***p < 0.001; BT, before the treatment; AT, after the treatment; Li-cit-5, group treated with 5 mM of lithium citrate; Li-cit-10, group treated with 10 mM of lithium citrate; OA, group treated with oxalic acid (positive control); C, negative control.

**FIGURE 7:**
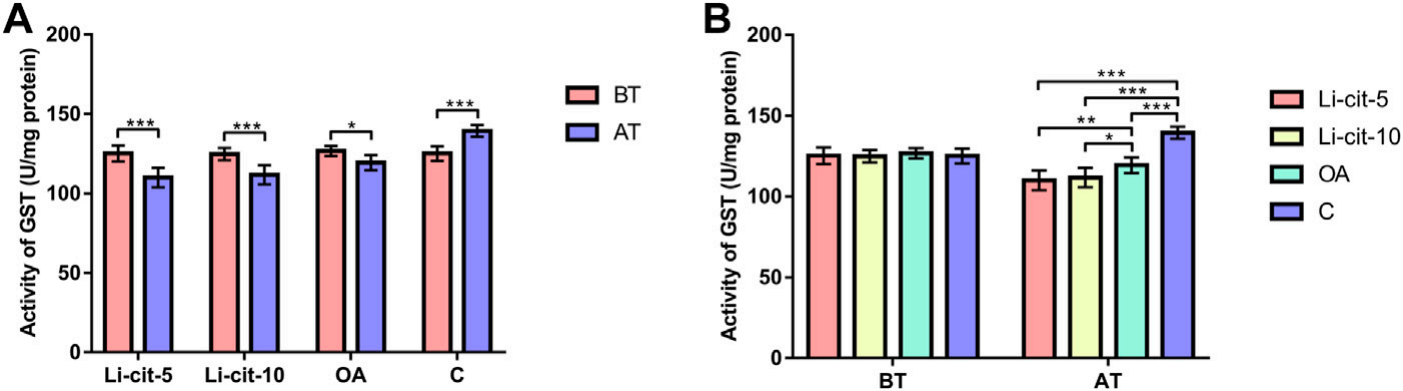
Glutathione-S transferase activity: comparisons between sampling occasions (before and after the treatment) within each group **(A)** and comparisons between groups at each sampling occasion **(B)**. Bars indicate mean ± standard deviation. *p < 0.05; **p < 0.01; ***p < 0.001; BT, before the treatment; AT, after the treatment; Li-cit-5, group treated with 5 mM of lithium citrate; Li-cit-10, group treated with 10 mM of lithium citrate; OA, group treated with oxalic acid (positive control); C, negative control.

**FIGURE 8:**
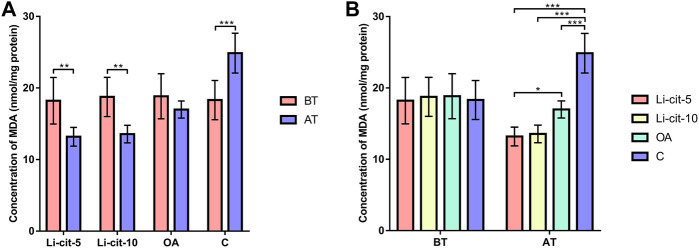
Malondialdehyde concentration: comparisons between sampling occasions (before and after the treatment) within each group **(A)** and comparisons between groups at each sampling occasion **(B)**. Bars indicate mean ± standard deviation. *p < 0.05; **p < 0.01; ***p < 0.001; BT, before the treatment; AT, after the treatment; Li-cit-5, group treated with 5 mM of lithium citrate; Li-cit-10, group treated with 10 mM of lithium citrate; OA, group treated with oxalic acid (positive control); C, negative control.

In Li-cit-5 and Li-cit-10 groups, the values of all monitored oxidative stress parameters were significantly lower (*p* < 0.01 or *p* < 0.001) after the treatment compared to pre-treatment values (Figures 5A, 6A, 7A, 8A).

In OA group, SOD, CAT and GST activities were significantly lower (*p* < 0.05 or *p* < 0.001) after the treatment compared to those recorded before the treatment (Figures 5A, 6A, 7A). On the contrary, in C group, CAT and GST activities and MDA concentration were significantly higher (*p* < 0.001) after the treatment (Figures 6A, 7A, 8A).

When the results were compared between groups before the treatment, no significant difference was found. Conversely, after the treatment, the values of all oxidative stress parameters were significantly higher (*p* < 0.001) in C group compared to Li-cit-5, Li-cit-10 and OA groups (Figures 5B, 6B, 7B, 8B). In OA group, GST activity was significantly higher (*p* < 0.05 or *p* < 0.01) compared to Li-cit-5 and Li-cit-10 groups (Figure 7B), CAT activity was significantly higher (*p* < 0.05) than in Li-cit-10 group (Figure 6B) while MDA concentration was significantly higher (*p* < 0.05) than in Li-cit-5 group (Figure 8B).

## Discussion

To our knowledge, this is the first study in which Li-cit was tested for its contact varroacidal efficacy and its influence on honey bees (by estimating their infestation with viruses and transcript levels of their immune-related genes and genes for antioxidative enzymes as well as bees’ oxidative stress). Concentrations of 5 and 10 mM were applied in the same way as oxalic acid (by trickling) and achieved the efficacy of 96.85% and 96.80%, respectively. So far, only Stanimirovic et al. (2022b) examined efficacy of Li-cit against *Varroa* on full-sized hives, but applied *via* feeding. In that study, varroacidal efficacy of tested Li-cit concentrations (5, 10, 15, 20, and 25 mM) ranged from 93.2 to 95.5%. A comparison of the above results indicates that Li-cit achieves greater contact than systemic varroacidal efficacy. Greater efficiency can be explained by the fact that our previous experiment (Stanimirovic et al., 2022b) was performed in period when the brood was present, while the current one was accomplished in broodless period. Another difference was the method of application. In the previous experiment, Li-cit was applied in 30% w/v sucrose solution in the summer period, three times in 6-day intervals in the amount of 1 L per colony. Mutual for the previous and this current experiment is that the follow-up treatments were carried out using CheckMite+^®^.

Li-cit in this study was tested in the same way as Li chloride in studies of Kolics et al. (2021a, 2022), by trickling lithiated sugar syrup in broodless period. However, varroacidal efficacy (96.85%–96.80%) we achieved by single treatment of low concentrations (5 and 10 mM) of Li-cit was slightly higher than the efficacy of 95.6% that Kolics et al. (2021a) achieved with much higher concentration (250 mM) of Li choride (also by single treatment). However, in most recent work of Kolics et al. (2022) only repeated trickling treatments with a 500 mM of Li chloride provided >90% efficiency. Based on the results of Kolics et al. (2021a, 2022) and the results of current study, it seems that the efficacy of Li salts is rather dependent on the mode of application (feeding vs. trickling treatment) than on concentration. On the contrary, in a work of Abd El-Wahab et al. (2021) efficiency of two ‘hard’ varroacides, Vapcozin-20 (amitraz) and Mavrik 2F (fluvalinate), was not dependent on the mode of application. In study of Kolics et al. (2021a), follow-up treatment was done with oxalic acid. In our current study, oxalic acid showed significantly lower efficacy (95.30%) than either of tested concentrations (5 and 10 mM) of Li-cit (96.85% and 96.80%, respectively). The appliance of oxalic acid in *Varroa* control has been extensively investigated (Lodesani et al., 2014; Giacomelli et al., 2016; Gregorc et al., 2016; Maggi et al., 2017), and it is known that the success of the treatment mostly depends on the period of the year, the presence of brood and the method of application. Oxalic acid exhibits the greatest efficacy during broodless periods (Gregorc and Planinc 2001; Gregorc et al., 2016) for the reason than it does not kill *Varroa* mites inside capped brood cells. Nevertheless, oxalic acid is relatively often used once a week for up to 3 weeks in period when the brood is present in the hive (Gregorc and Planinc 2001; Jack and Ellis, 2021), alone or in combination with brood interruption (Jack et al., 2021b). In this study, we recorded the efficacy of oxalic acid of 95.30% with only one trickling treatment, while Gregorc et al. (2016) in broodless colonies (obtained by caging and isolating queens) achieved only 24% of *Varroa* mortality with initial oxalic acid treatment, but three additional autumn oxalic acid treatments resulted in 97% varroacidal efficacy.

In both Li-cit treated groups in our study, loads of all monitored viruses (DWV, ABPV, CBPV and SBV) mainly decreased, significantly only in case of CBPV in Li-cit-5 group, after the treatment compared to the results affirmed before the treatment. In negative control (C group), loads of all viruses were significantly (*p* < 0.01 or *p* < 0.001) higher after the treatment. However, it is interesting that in OA group, loads of ABPV and DWV significantly (*p* < 0.05) rose, in spite of decrease of *Varroa* population. Similar findings were reported by Al Naggar et al. (2015) who evidenced decreased *Varroa* infestation level but not decrease in DWV infection rate after the treatment with Apivar (amitraz) and Thymovar (thymol). These results are in agreement with the report of Highfield et al. (2009) that DWV may persist independently of *Varroa* infestations. Besides, in spreading viral infections, other factors, such as virus contamination of food, virulence of the DWV and ABPV, horizontal transfer of infection from bee to bee, could also play roles (de Miranda et al., 2011; Locke et al., 2012; Stanimirović et al., 2019; Stanimirovic et al., 2022a). Another explanation of the significant increase in ABPV and DWV level in our study could be related with recorded changes in the expression levels of some immune-related genes. In fact, transcript levels of genes for abaecin and apidaecin (in Li-cit-10 group) and vitellogenin (in Li-cit-5 group) significantly (*p* < 0.01) increased over time, with simultaneous decrease of *Varroa* infestation level, and viruses loads too, compared to negative control (where transcript levels of all immune-related genes did not change significantly). It is worth to emphasize that transcript level of gene coding vitellogenin was significantly higher (*p* < 0.05) after the treatment only in Li-cit-5 group. Dainat et al. (2012) reported the decrease of vitellogenin gene expression level caused by DWV infection. *Varroa* parasitism had been reported to cause development of worker bees with decreased haemolymph proteins including vitellogenin (Amdam et al., 2004), that is considered as a biomarker for winter bee longevity (Dainat et al., 2012). In study of Smart et al. (2016) higher levels of vitellogenin gene transcripts in September were related to improved overwinter survival, so our finding may indicate positive influence of the autumn treatment with 5 mM of Li-cit on survival of colonies during winter. Along with vitellogenin, defensin is also important marker of overwinter survival (Smart et al., 2016), beside its role in bee immunity (Glavinic et al., 2017; Glavinic et al., 2019; Tesovnik et al., 2020; Glavinic et al., 2021a; Glavinic et al., 2021b; Glavinic et al., 2022). Barroso-Arévalo et al. (2019) reported correlation between decreased defensin gene expression and poor resistance to complex DWV-*Varroa*. In our study, both Li-cit treatments (5 and 10 mM) significantly knocked down *Varroa* mites with a simultaneous increase in defensin gene expression, suggesting stimulative effect of Li-cit on the resistance of bees to *Varroa* and DWV. On the contrary, in colonies treated with oxalic acid and those from the control group, defensin gene transcript level did not change. Nevertheless, in OA group, there were significantly increased expression levels of abaecin and apidaecin genes after the treatment, which may have reflected in significant increase of ABPV and DWV loads, also recorded in that group.

In both Li-cit groups in our study, the treatments did not lead to a significant change in expression of genes for antioxidant enzymes. It should be noted that Aurori et al. (2014) reported that the increase in the expression level of antioxidant enzymes occurs only in January (the middle of the overwintering period), and our experiment was performed in November. Interestingly, in our study, the activities of antioxidant enzymes SOD, CAT and GST significantly decreased after the treatment. Furthermore, the concentration of MDA also significantly decreased in both Li-cit groups, suggesting positive effect of Li-cit treatment by decreasing oxidative damage of cell membrane lipids in treated bees. This finding is probably the consequence of varroacidal effect of Li-cit and also its reducing effect on *Varroa*-associated viruses. In negative control (C group) of this study, after the treatment, significant increase in transcript levels of genes for CuZnSod, MnSOD and GST was recorded, along with an increase in antioxidant activity of CAT and GST and in MDA concentration. Previously, increased antioxidative enzyme activities in honey bees were reported as consequence of influence of *Varroa* infection (Badotra et al., 2013), which probably was also the case in our negative control. Other pathogens could have also contributed to the increase of enzyme activities, as *Nosema ceranae* did in Glavinic et al. (2017), Glavinic et al. (2021b), Glavinic et al. (2022) and other microbial pathogens depending on type of beekeeping (Taric et al., 2019, 2020).

On the other hand, in OA group (similar to the situation in Li-cit groups), none of the genes for antioxidative enzymes had significantly changed expression over time. However, the activities of antioxidant enzymes in OA group after the treatment, although lower compared to pre-treatment state, were higher compared to those recorded in Li-cit groups. The concentration of MDA in OA group did not differ significantly between the beginning and the end of the experiment. These changes in antioxidant protection might be associated with the sublethal effects of oxalic acid, knowing that oxalic acid may remain in the hive for more than 2 weeks after the end of the treatment (Rademacher et al., 2017). Shortly after the treatment with oxalic acid, Rademacher et al. (2017) noticed pronounced self-grooming in bees and thus the intake of acid *via* sucrose syrup. This behavior of bees (self-grooming) could have also been elicited by oxalic acid in this study and might contributed to the spread of DWV and ABPV and increase in their loads (Grozinger and Flenniken 2019; Stanimirović et al., 2019).

In this regard, the changes that occur in the organisms of bees are reflected in the increased demand for water intake and in the increase of acidity in the digestive tract and hemolymph. Consequently, oxalic acid leads to damage of the epithelial cells of bee digestive tract (Gregorc and Škerl, 2007; Rademacher et al., 2017), which leads to an increased activity of antioxidant enzymes and an increase in MDA concentration in their organisms. Similar effects were reported for coumaphos which, applied in the winter treatment of varroosis, induced changes in parameters of oxidative stress (Zikic et al., 2020).

## Conclusion

The results of this study: 1) confirmed great contact varroacidal efficacy of low concentrations (5 and 10 mM) of Li-cit; 2) revealed positive effect of Li-cit on honey bees based on measurements of immune-gene expression and oxidative stress markers; 3) showed negative effect of OA based on its influence on virus loads, but also lower efficacy in decreasing oxidative stress in bees compared to Li-cit treatments. The results related to the contact effects of Li-cit should be taken into account in development of a new formulation for *Varroa* control. However, before Li-cit trickling method becomes common practice for *Varroa* control, additional research is necessary to be performed in other regions and under different conditions, together with monitoring of development and behaviour of bees and assessment of residue levels in honey, wax and other hive products.

## Data Availability

The original contributions presented in the study are included in the article/Supplementary Material, further inquiries can be directed to the corresponding author.
