# Supplementary material for: Contact varroacidal efficacy of lithium citrate and its influence on viral loads, immune parameters and oxidative stress of honey bees in a field experiment
Source: Front Physiol. 2022 Sep 12;13:1000944. doi: 10.3389/fphys.2022.1000944 (PMC9510912; doi:10.3389/fphys.2022.1000944)
Supplement: Supplementary file 3 [file Table1.DOCX]

**Supplementary Table 1. Primers used in study**

| **Primer** | **Gene description** | **5'→3'** | **Reference** |
| --- | --- | --- | --- |
| ABPV-F | Acute bee paralysis virus | TCCTATATCGACGACGAAAGACAA | Chantawannakul et al. 2006 |
| ABPV-R |  | GCGCTTTAATTCCATCCAATTGA |  |
| DWV-F | Deformed wing virus | CCTGGACAAGGTCTCGGTAGAA | Chantawannakul et al. 2006 |
| DWV-R |  | ATTCAGGACCCCACCCAAAT |  |
| SBV-F | Sacbrood virus | AAGTTGGAGGCGCGYATTTG | Chantawannakul et al. 2006 |
| SBV-R |  | CAAATGTCTTCTTACDAGAAGYAAGGATTG |  |
| CBPV-F | Chronic bee paralysis virus | CGCAAGTACGCCTTGATAAAGAAC | Blanchard et al. 2007 |
| CBPV-R |  | ACTACTAGAAACTCGTCGCTTCG |  |
| Abaecin-F | Abaecin, antimicrobial peptide | CAGCATTCGCATACGTACCA | Evans et al. 2006 |
| Abaecin-R |  | GACCAGGAAACGTTGGAAAC |  |
| ApidNT-F | Apidaecin, antimicrobial peptide | TTTTGCCTTAGCAATTCTTGTTG | Simone et al 2009 |
| ApidNT-R |  | GTAGGTCGAGTAGGCGGATCT |  |
| Defensin-F | Defensin, antimicrobial peptide | TGCGCTGCTAACTGTCTCAG | Evans et al. 2006 |
| Defensin-R |  | AATGGCACTTAACCGAAACG |  |
| Hymenopt-F | Hymenoptaecin antimicrobial peptide | CTCTTCTGTGCCGTTGCATA | Evans et al. 2006 |
| Hymenopt-R |  | GCGTCTCCTGTCATTCCATT |  |
| Vit-F | Vitellogenin | AGTTCCGACCGACGACGA | Simone et al 2009 |
| Vit-R |  | TTCCCTCCCACGGAGTCC |  |
| GST-F | Glutathion-S transferase | AGGAGAGGTGTGGAGAGATAGTG | Li et al. 2014 |
| GST-R |  | CGCAAATGGTCGTGTGGATG |  |
| Cu/ZnSOD-F | CuZn Superoxide dismutase | TCAACTTCAAGGACCACATAGTG | Li et al. 2014 |
| Cu/ZnSOD-R |  | ATAACACCACAAGCAAGACGAG |  |
| MnSOD-F | Mn Superoxide dismutase | GTCGCCAAAGGTGATGTCAATAC | Li et al. 2014 |
| MnSOD-R |  | CGTCTGGTTTACCGCCATTTG |  |
| CAT-F | Catalase | TTCTACTGTGGGTGGCGAAAG | Li et al. 2014 |
| CAT-R |  | GTGTGTTGTTACCGACCAAATCC |  |
| Beta actin-F | Beta actin, house keeping gene | TTGTATGCCAACACTGTCCTTT | Simone et al 2009 |
| Beta actin-R |  | TGGCGCGATGATCTTAATTT |  |

Reference list:

1. Blanchard, P., Ribière, M., Celle, O., Lallemand, P., Schurr, F., Olivier, V., Iscache, A.L. Faucon, J.P. (2007). Evaluation of a real-time two-step RT-PCR assay for quantitation of Chronic bee paralysis virus (CBPV) genome in experimentally-infected bee tissues and in life stages of a symptomatic colony. J. Virol. Methods 141, 7-13.
2. Chantawannakul, P., Ward, L., Boonham, N. and Brown, M., (2006). A scientific note on the detection of honeybee viruses using real-time PCR (TaqMan) in *Varroa* mites collected from a Thai honeybee (*Apis mellifera*) apiary. J. Invertebr. Pathol. 91, 69-73.
3. Evans, J.D., Aronstein, K., Chen, Y.P., Hetru, C., Imler, J.L., Jiang, H., Kanost, M., Thompson, G.J., Zou, Z., Hultmark, D. (2006). Immune pathways and defence mechanisms in honey bees *Apis mellifera*. Insect Mol. Biol. 15, 645-656.
4. Li, C., Xu, B., Wang, Y., Yang, Z., Yang, W. (2014). Protein content in larval diet affects adult longevity and antioxidant gene expression in honey bee workers. Entomol. Exp. Appl. 151, 19-26.
5. Simone, M., Evans, J.D., Spivak, M. (2009). Resin collection and social immunity in honey bees. Evol. Int. J. Org. Evol. 60, 3016-3022.
