# Supplementary material for: Contact varroacidal efficacy of lithium citrate and its influence on viral loads, immune parameters and oxidative stress of honey bees in a field experiment
Source: Front Physiol. 2022 Sep 12;13:1000944. doi: 10.3389/fphys.2022.1000944 (PMC9510912; doi:10.3389/fphys.2022.1000944)
Supplement: Supplementary file 4 [file DataSheet3.PDF]

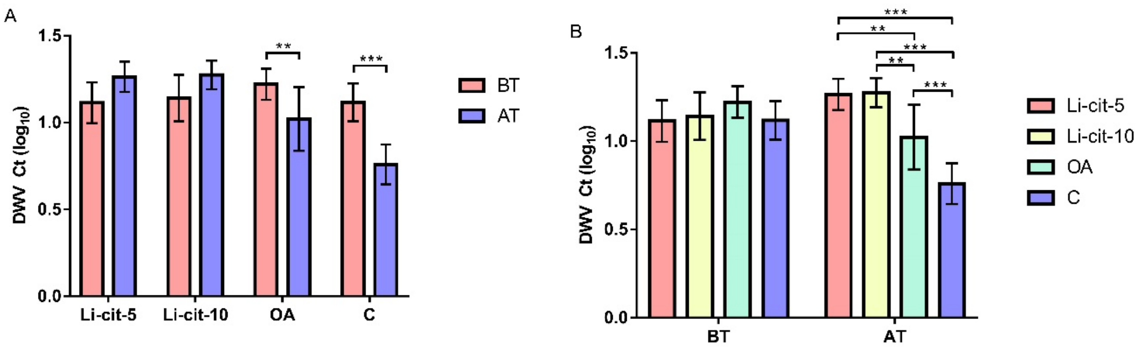

Supplementary Figure 3. Ct values (mean of Log10) of Deformed wing virus (DWV): comparisons between sampling occasions (before and after the treatment) within each group (A) and comparisons between groups at each sampling occasion (B). Bars indicate mean ± standard deviation. **p<0.01; ***p<0.001; cycle threshold, lower Ct indicates higher amount of viral RNA and vice versa for higher Ct; DWV, Deformed wing virus; BT, before the treatment; AT, after the treatment; Li-cit-5, group treated with 5 mM of lithium citrate; Li-cit-10, group treated with 10 mM of lithium citrate; OA, group treated with oxalic acid (positive control); C, negative control.
